# Oedema on STIR modified the effect of amoxicillin as treatment for chronic low back pain with Modic changes—subgroup analysis of a randomized trial

**DOI:** 10.1007/s00330-020-07542-w

**Published:** 2020-11-27

**Authors:** Per Martin Kristoffersen, Lars C. H. Bråten, Nils Vetti, Lars Grøvle, Christian Hellum, Kjersti Storheim, John-Anker Zwart, Jörg Assmus, Ansgar Espeland

**Affiliations:** 1 Department of Radiology, Haukeland University Hospital, Jonas Liesvei 65, 5021 Bergen, Norway; 2 Department of Clinical Medicine, University of Bergen, P.O. Box 7804, 5020 Bergen, Norway; 3 Research and Communication Unit for Musculoskeletal Health (FORMI), Oslo University Hospital HF, Ulleval, Bygg 37b, P.O. Box 4956, 0424 Oslo, Nydalen Norway; 4 Department of Rheumatology, Østfold Hospital Trust, P.O. Box 300, 1714 Grålum, Norway; 5 Division of Orthopaedic Surgery, Oslo University Hospital Ulleval, P.O. Box 4950, Nydalen, 0424 Oslo, Norway; 6 Faculty of Health Science, OsloMet - Oslo Metropolitan University, P.O. Box 4, St. Olavs plass, 0130 Oslo, Norway; 7 Faculty of Medicine, University of Oslo, P.O. Box 1072, Blindern, 0316 Oslo, Norway; 8 Competence Centre for Clinical Research, Haukeland University Hospital, Jonas Liesvei 65, 5021 Bergen, Norway

**Keywords:** Magnetic resonance imaging, Spine, Low back pain, Amoxicillin, Prospective studies

## Abstract

**Objective:**

To evaluate potential MRI-defined effect modifiers of amoxicillin treatment in patients with chronic low back pain and type 1 or 2 Modic changes (MCs) at the level of a previous lumbar disc herniation (index level).

**Methods:**

In a prospective trial (AIM), 180 patients (25–64 years; mean age 45; 105 women) were randomised to receive amoxicillin or placebo for 3 months. Primary outcome was the Roland-Morris Disability Questionnaire (RMDQ) score (0–24 scale) at 1 year. Mean RMDQ score difference between the groups at 1 year defined the treatment effect; 4 RMDQ points defined the minimal clinically important effect. Predefined baseline MRI features of MCs at the index level(s) were investigated as potential effect modifiers. The predefined primary hypothesis was a better effect of amoxicillin when short tau inversion recovery (STIR) shows more MC-related high signal. To evaluate this hypothesis, we pre-constructed a composite variable with three categories (STIR1/2/3). STIR3 implied MC-related STIR signal increases with volume ≥ 25% and height > 50% of vertebral body and maximum intensity increase ≥ 25% and presence on both sides of the disc. As pre-planned, interaction with treatment was analysed using ANCOVA in the per protocol population (*n* = 155).

**Results:**

The STIR3 composite group (*n* = 41) and STIR signal volume ≥ 25% alone (*n* = 45) modified the treatment effect of amoxicillin. As hypothesised, STIR3 patients reported the largest effect (− 5.1 RMDQ points; 95% CI − 8.2 to − 1.9; *p* for interaction = 0.008).

**Conclusions:**

Predefined subgroups with abundant MC-related index-level oedema on STIR modified the effect of amoxicillin. This finding needs replication and further support.

**Key Points:**

• *In the primary analysis of the AIM trial, the effect of amoxicillin in patients with chronic low back pain and type 1 or 2 MCs did not reach the predefined cut-off for clinical importance.*

• *In the present MRI subgroup analysis of AIM, predefined subgroups with abundant MC-related oedema on STIR reported an effect of amoxicillin.*

• *This finding requires replication and further support.*

**Supplementary Information:**

The online version contains supplementary material available at 10.1007/s00330-020-07542-w.

## Introduction

Modic changes (MCs) are magnetic resonance imaging (MRI) findings of vertebral bone marrow changes extending from the endplate. MCs are defined as type 1 (oedema type), 2 (fatty type), and 3 (sclerotic type) based on their intensity on T1- and T2-weighted MRI [1, 2]. However, the MC intensity and type depend on MRI scanning parameters and magnetic field strength [3, 4], and different MC types may represent stages of a common biological process [5]. This process may involve inflammation, fibrosis, high bone turnover, fatty infiltration, and sclerosis [1, 5, 6]. MCs were related to low back pain (LBP) in some studies but not in others [7–9]. The evidence for an association between MCs and LBP is stronger for type 1 than for type 2 or 3 MCs [10–15]. Proposed explanations for MCs include endplate damage, autoimmunity, and occult discitis [5]. Vertebral bone oedema resembling type 1 MCs is a common MRI finding in spondylodiscitis [16], and one theory is that MCs develop adjacent to a low-grade discitis region caused by haematogenic spread of *Cutibacterium acnes* bacteria to a previously disrupted, neo-vascularised lumbar disc [17, 18].

A previous trial reported a substantial effect of antibiotic treatment for chronic LBP with type 1 MCs on 0.2-T MRI [19]. Some of these MCs might have appeared as type 2 on 1.5-T MRI [4]. The recent AIM (Antibiotics In Modic changes) trial applied 1.5-T MRI [20] and reported a small but not clinically important effect of amoxicillin in chronic LBP patients with type 1 MCs (− 2.3 points on the Roland-Morris Disability Questionnaire (RMDQ)) and no effect for type 2 MCs (− 0.1 RMDQ points) [21]. Thus, the type 1 (oedema type) group tended to have a larger effect. More bone marrow oedema may be associated with worse pain and disability [22–24] and might indicate more severe disease with a larger potential for improvement. Therefore, we hypothesised a larger effect of amoxicillin in subgroups with more versus less MC-related oedema. It is relevant to assess subgroup effects across both MC types because both can contain inflammatory changes [25] and oedema [26].

Short tau inversion recovery (STIR) series are ideal for highlighting oedema. STIR suppresses a high signal from fat and often shows oedema in MCs classified as type 2 on T1/T2 series without fat suppression [26]. Although STIR is more sensitive to bone marrow oedema than standard T1/T2 series [26, 27], STIR can likewise not separate infectious from non-infectious causes [28, 29]. This subgroup study of the AIM trial included both STIR and standard fast spin echo T1/T2 sequences. We aimed to evaluate potential MRI-defined effect modifiers of amoxicillin treatment in patients with chronic LBP and type 1 or 2 MCs at the level of a previous lumbar disc herniation.

## Materials and methods

The AIM trial included 180 patients from six hospital outpatient clinics in Norway from June 2015 to September 2017 [20, 21]. All the eligibility criteria are detailed in the Appendix, Table A1. The inclusion criteria were age 18–65 years, LBP for more than 6 months with a mean intensity of at least 5 on three 0–10 numerical rating scales, lumbar disc herniation on MRI in the preceding 2 years, and type 1 or type 2 MCs (with height ≥ 10% of the vertebral height and diameter > 5 mm) at the previously herniated disc level. Trial flow chart, trial methods, and baseline characteristics are published [21]. The trial, this study, and statistical analysis plans are registered at ClinicalTrials.gov (identifier: NCT02323412).

### Randomisation, treatment, and outcome measures

Patients were randomised to receive oral amoxicillin 750 mg or placebo (maize starch) three times daily for 3 months. The amoxicillin and placebo tablets had identical encapsulation, containers, and labelling. A third-party statistician used Stata 13 (StataCorp) to create randomisation lists. Allocation was stratified by prior disc surgery (yes/no) and MC type (type 1 (*n* = 118) or type 2 (*n* = 62) only) at the previously herniated disc level(s) with a 1:1:1:1 allocation and random block sizes of four and six [21]. The allocation sequence was concealed and centrally administered. Care providers gave patients a prescription with a computer-generated allocation number to be used at dedicated pharmacies. All care providers, research staff, statisticians, and patients were blinded to treatment allocation during data collection.

The primary endpoint was the RMDQ score (0–24 scale) at 1 year [30, 31]. The minimal clinically important difference in mean RMDQ score at 1 year between treatment groups (treatment effect) was predefined as 4 [20, 21]. The outcome at the end of the treatment period (3 months) was not primary because long-term improvement is desirable and the antibiotic group in the prior trial improved by 4.5 RMDQ points (0–23 scale) from the end of the treatment period to 1 year [19]. The Oswestry Disability Index (ODI) 2.0 (0–100 scale) [32] and LBP intensity (0–10 numeric rating scale) were secondary outcomes [21].

### MRI assessment

Baseline MRI of the lumbar spine was performed at six centres using identical protocols and the same type of 1.5-T scanner with the same software version (Magnetom Avanto B19; Siemens). This MRI included sagittal T1- and T2-weighted fast spin echo (‘T1/T2’) and sagittal STIR images. The integrated spine array coil was used, but no surface coils. Echo time (ms)/repetition time (ms) was 11/575 for T1, 87/3700 for T2, and 70/5530 for STIR. Echo train length was 5 for T1, 17 for T2, and 20 for STIR. Matrix was 384 × 269 for T1/T2 and 320 × 224 for STIR. The inversion time for STIR was 160 ms. Slice thickness/spacing was 4 mm/0.4 mm and field of view was 300 mm × 300 mm for all three sequences. Other MRI parameters were also identical between centres [33].

Three radiologists (A.E., N.V., and P.M.K.) who were blinded to clinical outcomes and treatment allocation independently rated MRI findings [33]. All had > 10 years of experience in musculoskeletal MRI. The same three radiologists interpreted the MRIs from all study centres. On T1/T2, they rated primary (most extensive) and secondary MC types as type 1 (hypointense on T1, hyperintense on T2), type 2 (hyperintense on T1, iso- or hyperintense on T2), and type 3 (hypointense on T1 and T2) [33]. They evaluated the largest height and volume of MCs on T1/T2 and the largest height, volume and intensity of any MC-related STIR signal increase (Table 1), defined as a visible increase compared with normal vertebral bone marrow, located in or abutting a region with MC on T1/T2 or located and shaped as an MC [33].

**Table 1:** Predefined MRI variables at index level(s) with type 1 or 2 MCs and prior disc herniation

	Description	Analysed subgroups
STIR signal extent and intensity		
STIR volume	Largest volume of high STIR signal in % of vertebral body marrow volume, visually estimated (not measured), and scored as 0, 1 (< 10%), 2 (< 25%), 3 (25–50%), or 4 (> 50%)	Scores 0–1, 2, and 3–4
STIR height	Maximum height of region with high STIR signal, measured and recalculated into a percentage of vertebral body marrow height	Analysed as a continuous variable and dichotomised into ≤ 50% and > 50%
STIR intensity	Maximum intensity of the high STIR signal, measured as a percentage on a scale from normal vertebral body marrow intensity (0%) to cerebrospinal fluid intensity (100%)	< 25%, 25–40%, and > 40%^a^
STIR sup/inf	Presence of high STIR signal both superior (sup) and inferior (inf) to index disc (yes/no)	Yes and no
STIR composite	Categorised as STIR3 (volume ≥ 25% AND height > 50% AND intensity ≥ 25% AND yes for sup/inf), STIR2 (not STIR3 or STIR1) and STIR1 (volume < 25% AND intensity < 25%)	STIR1, STIR2, and STIR3
MC type and extent on T1/T2		
MC type 1 degree	Categorised as type 1 major (primary type 1 both superior and inferior to disc), type 1 minor (primary or secondary type 1, but not type 1 major), and type 2 only (not type 1)	Type 2 only, type 1 minor, and type 1 major
MC volume	Largest volume of MC (including all MC types) in % of vertebral body marrow volume, visually estimated, and scored as 0, 1 (< 10%), 2 (< 25%), 3 (25–50%), or 4 (> 50%)	Scores 1, 2, and 3–4 (not score 0; conclusive MC volume cannot be 0 at an index level)
MC height	Maximum MC height (including all MC types), measured and recalculated as a percentage of vertebral body marrow height	Analysed as a continuous variable and dichotomised into ≤ 50% and > 50%
MC composite	Categorised as 1++ (type 1 major AND volume ≥ 10%—OR—primary type 1 AND volume ≥ 25% AND height ≥ 50% AND yes for sup/inf), 1+ (primary or secondary type 1 but not type 1++) and type 2 only	Type 2 only, 1+, and 1++

*MRI*, magnetic resonance imaging; *MCs*, Modic changes; *STIR*, short tau inversion recovery; *T1/T2*, T1- and T2-weighted fast spin echo images

^a^STIR intensity < 25% included MCs with no conclusive STIR signal increase (or decreased STIR signal) and thus no conclusive measured STIR intensity values. These values had likely been < 25% if measured, since the STIR intensity was < 20% for > 90% of intensity measurements reported by only one radiologist (as the other radiologists found no visual signal increase and therefore did not measure STIR intensity). To enhance clinical credibility, images were reviewed to ensure that intensity ≥ 25% implied visually convincing hyper-intensity

We based the conclusive MRI findings on the radiologists’ majority rating or mean value of measurements made by two of them (A.E. and P.M.K. if all three agreed there was a lesion to measure). The inter-rater reliability of the MRI evaluations was previously reported [33]. Fleiss’ kappa values [34] for overall inter-rater agreement (mean values across four endplates L4-S1) were 0.88/0.81 for presence of any MCs/type 1 MCs, 0.64/0.69 for MC height/volume, 0.86 for presence of a STIR signal increase, and 0.51/0.56 (0.40/0.40 at L5/S1 inferior to disc) for STIR signal height/volume. For maximum MC-related STIR signal intensity on a 0–100% scale (0% = normal vertebral body; 100% = cerebrospinal fluid), largest mean of differences and widest 95% limits of agreement were 0.9% and ± 7.6%, respectively.

### Predefined hypotheses and potential effect modifiers

In the AIM trial protocol, we hypothesised a better effect of amoxicillin when:

- STIR shows more MC-related high signal (primary hypothesis)
- MCs contain more type 1 than type 2 or are larger (explorative hypothesis)

The nine variables described in Table 1 were predefined as potential effect modifiers in the statistical analysis plan. All concerned MCs at the index level(s) with prior disc herniation because this level was hypothesised to contain low-grade discitis that was the target for treatment. One composite and four underlying variables concerned STIR signal extent and intensity. The STIR composite variable had three categories (STIR1/2/3) and was used to assess the primary hypothesis. STIR3 implied MC-related STIR signal increase with volume ≥ 25% and height > 50% of the vertebral body, maximum intensity increase ≥ 25%, and presence on both sides of the disc. One composite and three underlying variables concerned MC extent and type 1 degree on T1/T2. We constructed each composite variable by clinically plausible grouping of the underlying variables [35]. We did so before analysing the effect of any MRI variable on the outcome but were not blinded to the distribution of the variables in our sample.

### Analyses

The baseline properties of the randomised groups were characterised using descriptive methods.

All pre-planned analyses are described in Table 2. Each effect modifier was analysed using ANCOVA with the outcome (RMDQ, ODI, or LBP intensity) at 1 year as the dependent variable and the randomisation group, effect modifier, and their interaction as independent variables adjusted for the baseline values of the outcome. Additionally, we adjusted for age and prior disc surgery because contamination during surgery is a potential cause of discitis. Supporting the use of ANCOVA, Levene’s test indicated homogeneity of variances, and QQ plots indicated normally distributed residuals and outcome variables without extreme outliers. If one or both composite MRI variables modified the treatment effect, we would include both in the same model to assess their independence [36]. Post hoc analyses, marked as such throughout the manuscript, are also described in Table 2.

**Table 2:** Analyses—pre-planned and post hoc

	Analysed population
Pre-planned analyses	
ANCOVA^a^—dependent variable: RMDQ score at 1 year; independent variables: baseline RMDQ score, the potential effect modifier, treatment group, their interaction term (effect modifier^a^ treatment), age, former disc herniation surgery	PP and ITT
ANCOVA^a^—as above with ODI score replacing RMDQ score	PP and ITT
ANCOVA^a^—as above with LBP intensity score replacing RMDQ score	PP and ITT
ANCOVA^a^—to assess the independency of any effect modification for STIR composite and/or MC composite; dependent variable: RMDQ score at 1 year; independent variables: baseline RMDQ score, STIR composite, MC composite, age, treatment, STIR composite*treatment, MC composite*treatment, age*treatment, former disc herniation surgery	PP
Post hoc analyses—to evaluate STIR3 results	
Comparison of treatment groups—baseline factors, concomitant treatment; no statistical testing	STIR3 group
Responder analyses—number needed to treat to achieve > 30%, > 50%, and > 75% reduced RMDQ score from baseline to 1 year (for those with complete data)	STIR3 group with data
Distribution of responders—number and proportions of patients with > 30%, > 50%, and > 75% reduced RMDQ score at 1 year (those with complete data) by treatment group and STIR group	All with data
Linear mixed-effects models—to assess treatment effect over time; using Akaike’s information criterion to decide which covariance matrix to apply; dependent variable: RMDQ (5 time points), ODI (3 time points), or LBP intensity (17 time points); independent variables: time, treatment, time*treatment, age, prior disc herniation surgery	PP STIR3 group
Scatterplot of change in RMDQ score—to visualise change in RMDQ score from baseline to 1 year in each treatment group for STIR1, STIR2, and STIR3 patients (those with complete data)	PP with data
Bangs blinding index—for each treatment group based on their response at 1 year to ‘Which study medicine do you think you received?’ (antibiotics/placebo/unsure); range: − 1 (all report incorrect treatment) to 1 (all report the correct treatment); 0 = random reporting of treatment	STIR3 group

*PP*, per protocol; *ITT*, intention to treat; *RMDQ*, Roland-Morris Disability Questionnaire; *ODI*, Oswestry Disability Index; *LBP*, low back pain; *STIR*, short tau inversion recovery; *MC*, Modic change

^a^Missing values of RMDQ, ODI, or LBP intensity were substituted with imputed values from the multiple imputations performed in the trial. This multiple imputation model used 50 imputations, predictive mean matching, and the following predictors: age, leg pain, comorbidity, fear avoidance, emotional distress, physical workload, former surgery for disc herniation, study centre, MC type group, and treatment group

The primary analyses were performed on the predefined per protocol (PP) population described in the Appendix, page 2. The intention to treat (ITT) population was used for supportive analyses. Missing outcome values were imputed using multiple imputation (details in footnote, Table 2).

We used a Bonferroni-corrected alpha of 0.05/6 (0.008) when testing the primary hypothesis (ranked as hypothesis six in the trial protocol) [20]. Otherwise, an alpha of 0.05 was applied to minimise type 2 errors [37]. Analyses were performed using Stata 16 (StataCorp), and figures were made using MATLAB 9.5 (MathWorks) or Stata 16.

### Power calculation

The AIM trial was designed with 90% power in each MC type group [20]; 80% power in the total sample would have required 50 patients or 200 patients in a subgroup study with two equally large subgroups [38]. In this study with three subgroup categories, 80% power would have required > 200 patients. Adding covariates in the analyses improved the power [39], but our sample was still small.

## Results

**Fig. 1:**
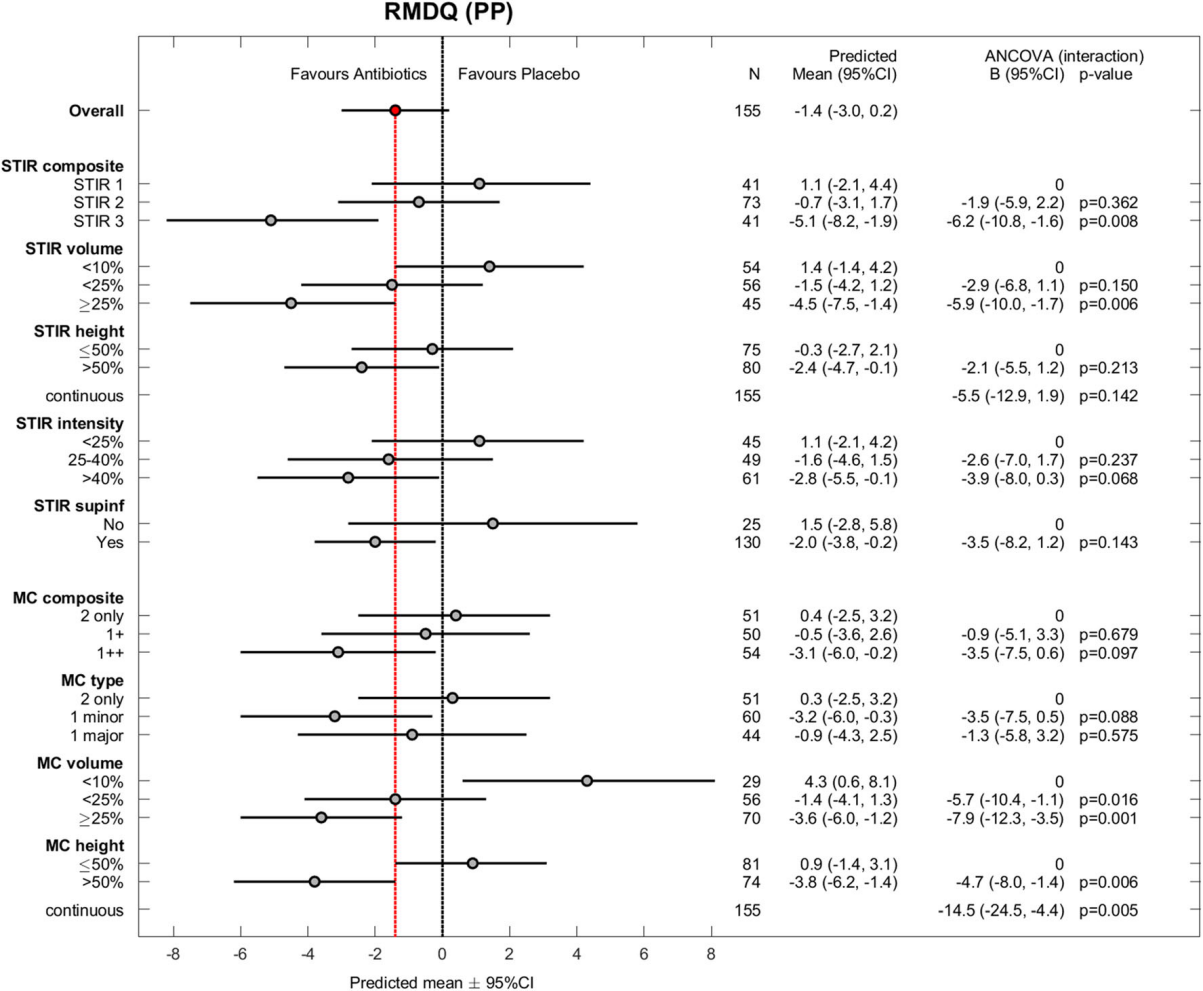
Roland-Morris Disability Questionnaire (RMDQ) for all effect modifiers (per protocol). RMDQ scores range from 0 (no disability) to 24 (maximum disability). Observed difference between treatment groups (mean ± 95% CI) and estimated coefficients (with 95% CI) for interaction from the ANCOVA (per protocol) with *p* values. PP, per protocol; CI, confidence interval; STIR, short tau inversion recovery; MC, Modic change. MC variables are based on T1- and T2-weighted fast spin echo images, not STIR

**Fig. 2:**
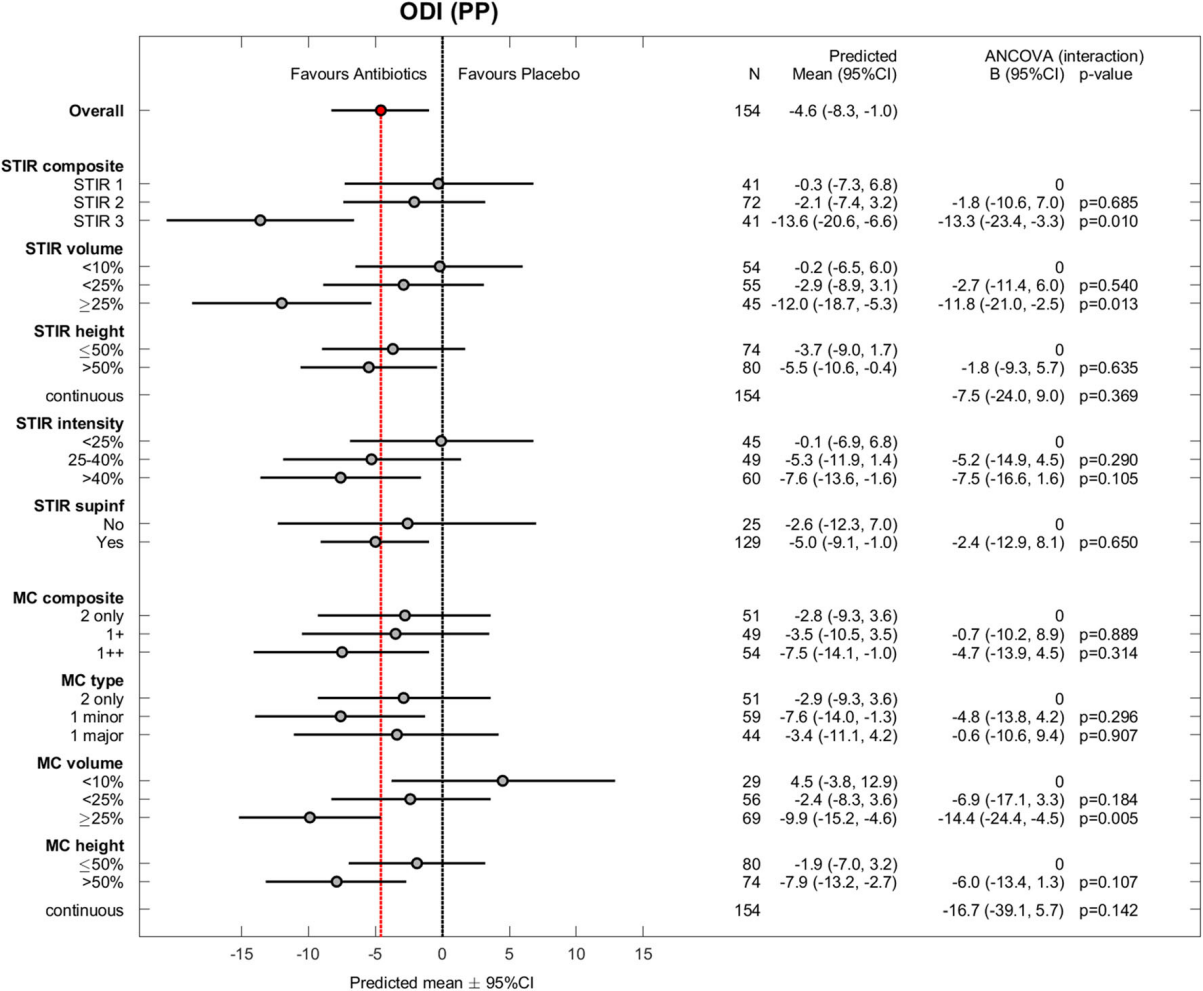
Oswestry Disability Index (ODI) for all effect modifiers (per protocol). ODI scores range from 0 (no disability) to 100 (maximum disability). Observed difference between treatment groups (mean ± 95% CI) and estimated coefficients (with 95% CI) for interaction from the ANCOVA (per protocol) with *p* values. Missing value not imputed in one patient (excluded). PP, per protocol; CI, confidence interval; STIR, short tau inversion recovery; MC, Modic change. MC variables are based on T1- and T2-weighted fast spin echo images, not STIR

**Fig. 3:**
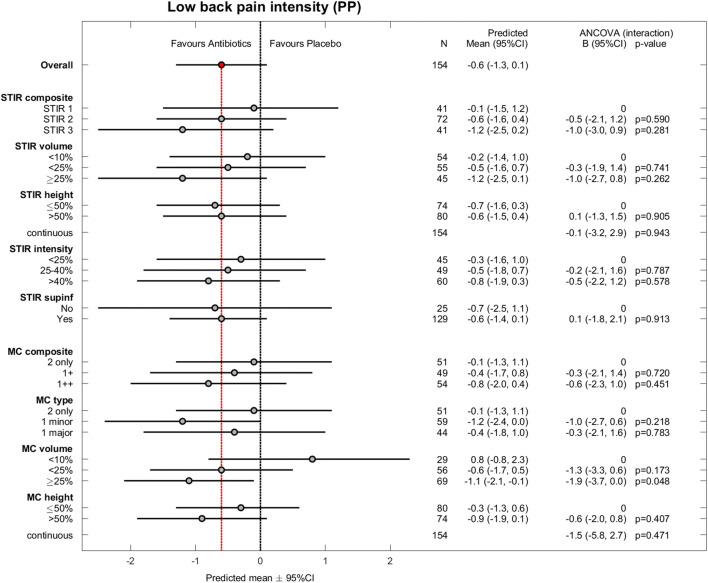
Low back pain intensity for all effect modifiers (per protocol). Pain intensity scores range from 0 (no pain) to 10 (worst possible pain). Observed difference between treatment groups (mean ± 95% CI) and estimated coefficients (with 95% CI) for interaction from the ANCOVA (per protocol) with *p* values. Missing value not imputed in one patient (excluded). PP, per protocol; CI, confidence interval; STIR, short tau inversion recovery; MC, Modic change. MC variables are based on T1- and T2-weighted fast spin echo images, not STIR

The 180 patients were aged 25–64 years (mean age 45 years; standard deviation 9 years); 105 (58%) patients were women. Table 3 shows baseline MRI findings by treatment group. Of 360 baseline and 1-year outcome values, 13 were missing for RMDQ, 14 for ODI, and 13 for LBP intensity. The results for the effect modifiers were similar in PP analyses (*n* = 155) (Figs. 1, 2, and 3) and ITT analyses (*n* = 180) (Appendix, Figs. A1–A3).

**Table 3:** Baseline index level MRI findings by treatment group in the total sample (*N* = 180)

	Amoxicillin group (*N* = 89)		Placebo group (*N* = 91)	
Variable	*n*	%	*n*	%
STIR composite				
STIR1	23	25.8	25	27.5
STIR2	42	47.2	45	49.5
STIR3	24	27.0	21	23.1
STIR volume—maximum score (% of vertebral body marrow volume; visually estimated)				
0 (0%)	11	12.4	5	5.5
1 (< 10%)	21	23.6	27	29.7
2 (< 25%)	31	34.8	36	39.6
3 (25–50%)	21	23.6	18	19.8
4 (> 50%)	5	5.6	5	5.5
STIR height—% of vertebral body marrow height				
Median (interquartile range)	51 (32–63)		51 (38–62)	
< 25	15	16.9	11	12.1
25–50	28	31.5	34	37.4
> 50	46	51.7	46	50.5
STIR intensity—% increase from normal vertebral body intensity (0%) to CSF intensity (100%)				
Mean (SD)	39 (14)		35 (14)	
< 25	25	28.1	27	29.7
25–40	26	29.2	31	34.1
> 40	38	42.7	33	36.3
STIR sup/inf—STIR signal increase both superior and inferior to disc				
Yes	72	80.9	78	85.7
No	17	19.1	13	14.3
MC composite				
Type 2 only	31	34.8	31	34.1
1+	19	21.3	36	39.6
1++	39	43.8	24	26.4
MC type I degree—categories				
Type 2 only	31	34.8	31	34.1
Type 1 minor	24	27.0	43	47.3
Type 1 major	34	38.2	17	18.7
MC volume—maximum score (% of vertebral body marrow volume; cannot be 0%)				
1 (< 10%)	17	19.1	15	16.5
2 (< 25%)	33	37.1	35	38.5
3 (25–50%)	30	33.7	29	31.9
4 (> 50%)	9	10.1	12	13.2
MC height—% of vertebral body marrow height				
Mean (SD)	51 (16)		49 (16)	
< 25	5	5.6	6	6.6
25–50	39	43.8	45	49.5
> 50	45	50.6	40	44.0
Index level(s) with MC and previous disc herniation				
L2/L3	2	2.2	2	2.2
L3/L4	7	7.9	5	5.5
L4/L5	48	53.9	29	31.9
L5/S1	58	65.2	74	81.3

*MRI*, magnetic resonance imaging; *STIR*, short tau inversion recovery; *SD*, standard deviation; *MC*, Modic change. MC variables are based on T1- and T2-weighted fast spin echo images, not STIR

### Primary hypothesis—STIR

As hypothesised, the STIR3 group (*n* = 41) reported the largest effect of amoxicillin; the difference in mean RMDQ score at 1 year between those receiving amoxicillin and those receiving placebo (PP analysis) was − 5.1 (95% CI − 8.2 to − 1.9, *p* for interaction = 0.008) (Fig. 1). The corresponding difference was − 0.7 (95% CI − 3.1 to 1.7) in the STIR2 group and 1.1 (95% CI − 2.1 to 4.4) in the STIR1 group. The treatment effect in the STIR3 group was − 4.8 points (95% CI − 7.9 to − 1.8; *p* for interaction = 0.014) in the ITT analysis and − 4.5 RMDQ points (95% CI − 7.9 to − 1.1, *p* for interaction = 0.14) in the PP model including both composite MRI variables.

STIR volume ≥ 25% of the vertebral body (*n* = 45) also significantly modified the treatment effect (Fig. 1). In the STIR3 and STIR volume ≥ 25% groups, the effect of amoxicillin was > 4 RMDQ points (cut-off for clinical importance) and also evident for ODI (Fig. 2) but not for LBP intensity (Fig. 3).

### Explorative hypothesis—T1/T2

The results for the composite MC variable based on T1/T2 did not reach statistical significance or clinical importance (Fig. 1). Two underlying subgroups significantly modified the treatment effect in favour of amoxicillin: MC volume ≥ 25% and MC height > 50% of the vertebral body (Fig. 1). The treatment effect within these subgroups did not exceed the threshold for clinical importance.

### Post hoc analyses of STIR3

The baseline characteristics of the STIR3 patients were similar in both treatment groups (Appendix, Table A5). The STIR3 patients and total sample had similar mean baseline RMDQ scores (amoxicillin/placebo 12.8/12.6 vs. 12.7/12.8) and prior disc surgery rates (16% vs. 21%).

The number of STIR3 patients needed to be treated to achieve > 30% improved RMDQ score at 1 year was 3.1 (95% CI 1.7 to 27) (Appendix, Tables A2–A3). Among patients receiving amoxicillin, 6 of 22 STIR3 patients (27%) improved > 75% compared with 9 of 41 (22%) STIR2 patients and no STIR1 patients.

**Fig. 4:**
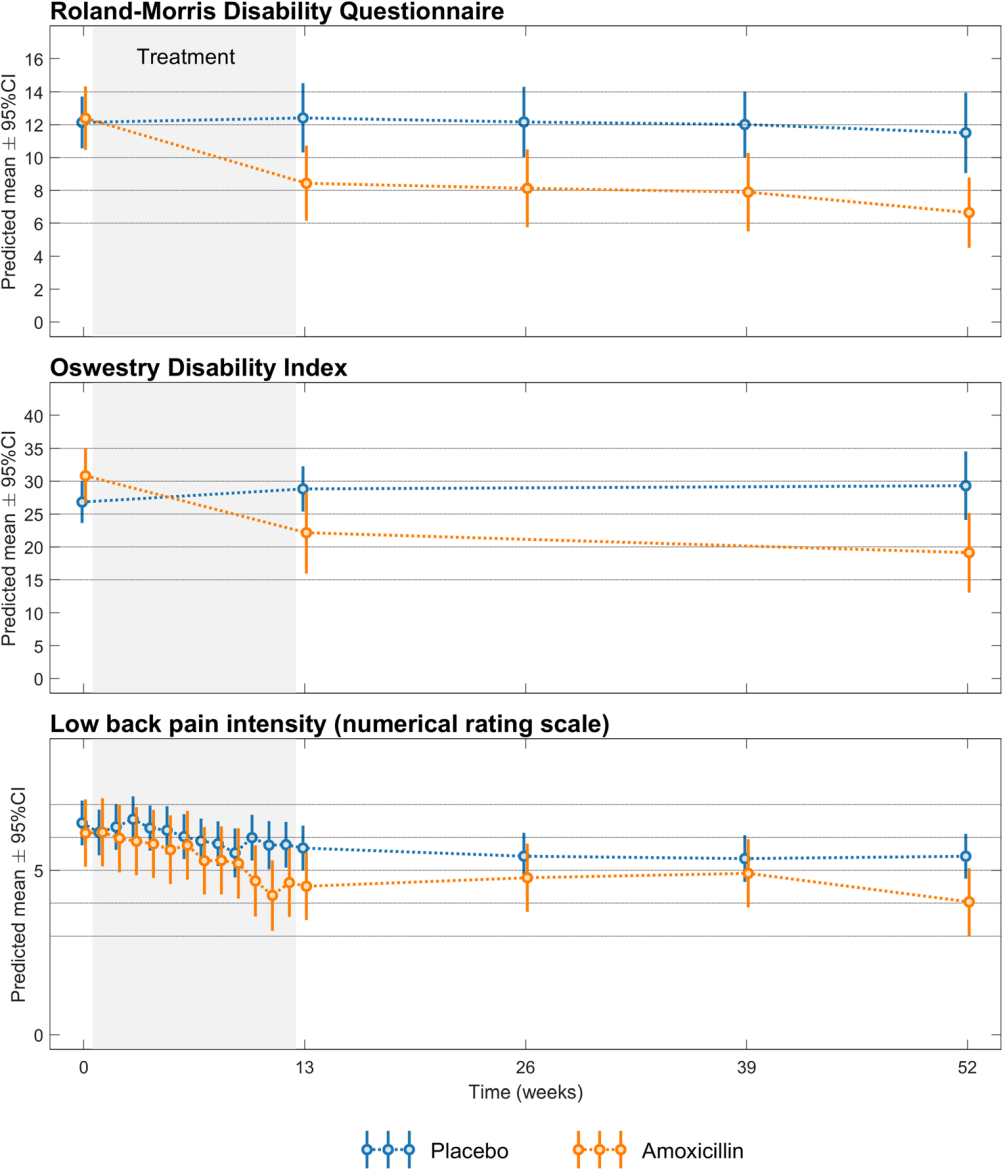
Treatment effect over time for STIR3 patients (per protocol). Roland-Morris Disability Questionnaire score (0–24 scale), Oswestry Disability Index score (0–100 scale), and low back pain intensity (0–10 numerical rating scale) from baseline to 1 year in each treatment group. Higher scores imply worse disability/pain. The results are from post hoc analyses

**Fig. 5:**
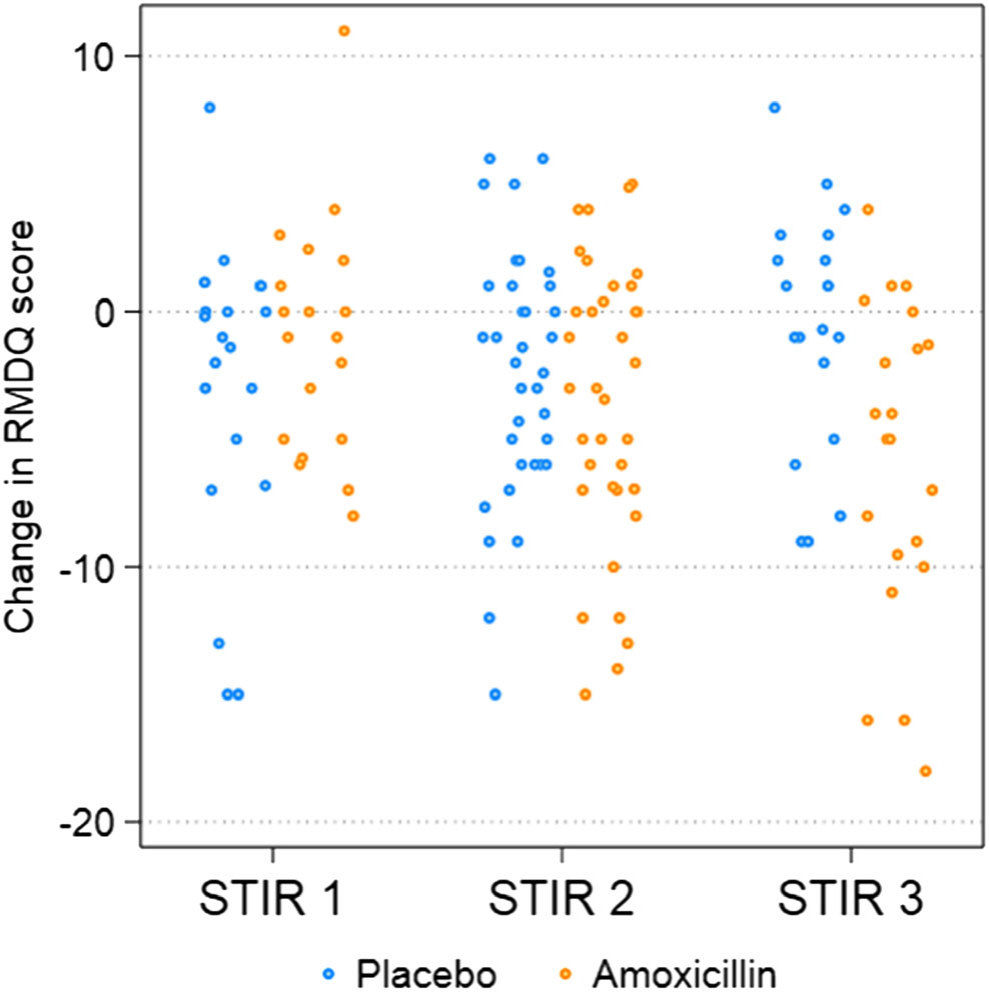
Change in the Roland-Morris Disability Questionnaire (RMDQ) score (per protocol). The change in the RMDQ score (0–24 scale) from baseline to 1 year in each treatment group is plotted for STIR1, STIR2, and STIR3 patients; negative values denote improvement. The results are from post hoc analyses. STIR, short tau inversion recovery

The treatment effect of amoxicillin in the STIR3 group was present at 3 months and remained until the end of the study at 1 year (Fig. 4), but the change in RMDQ score varied considerably between patients (Fig. 5).

Bangs blinding index for STIR3 patients was − 0.01 in the amoxicillin group, indicating perfect blinding, and 0.61 in the placebo group, indicating un-blinding (Appendix, Table A4).

## Discussion

To our knowledge, this was the first study to investigate STIR-based effect modifiers of a treatment for chronic LBP with MCs. These effect modifiers were defined by MC-related high signal on STIR at the previously herniated index level(s) hypothesised to contain a low-grade discitis. The STIR3 and STIR volume ≥ 25% groups with abundant high signal modified the treatment effect of amoxicillin. STIR3 patients reported the largest effect (− 5.1 RMDQ points; 95% CI − 8.2 to − 1.9; *p* for interaction = 0.008). Subgroups based on T1/T2 features of MCs did not report a clinically important effect of amoxicillin. All subgroups were small, and the findings must be interpreted with caution.

### Credibility of results

We consider the subgroup effect of STIR3 to have overall moderate credibility based on the criteria predefined in the statistical analysis plan [36] and the results of the post hoc analyses, but some criteria were not fulfilled (Table 4). The interaction of STIR3 and STIR volume ≥ 25% with treatment was found for the related outcomes RMDQ and ODI but not for LBP, and the finding has not yet been replicated in other studies. No tissue samples were taken, and STIR findings alone are not diagnostic for infection [28, 29]. Further data are needed to link extensive oedema on STIR to low-grade disc infection.

**Table 4:** Credibility of the subgroup effect of STIR3 (per protocol)

Criterion/evaluation	Comment
(1) Is the subgroup variable a characteristic measured at baseline or after randomisation?	Yes, at baseline.
(2) Is the effect suggested by comparisons within rather than between studies?	Yes, within this study.
(3) Was the hypothesis specified a priori?	Yes, in the trial protocol and statistical analysis plan
(4) Was the direction of the subgroup effect specified a priori?	Yes, and the a priori specified direction was verified
(5) Was the subgroup effect one of a small number of hypothesised effects tested?	Yes, the hypothesis was predefined as primary hypothesis for this study and pre-ordered as hypothesis six (F) in trial protocol
(6) Does the interaction test suggest a low likelihood that chance explains the apparent subgroup effect?	Yes, *p* = 0.008, equal to the Bonferroni adjusted alpha of 0.05/6; this strengthened the result, as the six tests were not independent
(7) Is the significant subgroup effect independent?	Probably. The subgroup effect only changed from 5.1 to 4.5 RMDQ points when analysing its independence
(8) Is the size of the subgroup effect large?	Yes, 5.1 RMDQ points (predefined minimal clinically important value was 4), but its 95% CI was wide (1.9 to 8.2)
(9) Is the interaction consistent across studies?	Not clear, no other study has examined this interaction
(10) Is the interaction consistent across closely related outcomes within the study?	Partly, consistent across the closely related disability outcomes RMDQ and ODI, but not significant for LBP intensity
(11) Is there indirect evidence that supports the hypothesised interaction (biological rationale)?	Partly. Discitis can cause MCs, type 1 MCs can be symptomatic, amoxicillin tended to reduce disability in patients with type 1 MCs (oedema) (see text). STIR3 group (extensive oedema) improved most during antibiotic treatment, like verified *Cutibacterium acnes* discitis (see text). No tissue samples taken to verify infection. Further evidence (e.g. biological) needed to link extent of oedema on STIR to likeliness of disc infection
Comparison of treatment groups	STIR3 treatment groups were similar in clinical baseline characteristics and in concomitant treatment during study, supporting the subgroup effect
Responder analyses	STIR3: NNT 3–4 to achieve > 30–75% improved RMDQ from baseline to 1-year follow-up, but wide CIs
Treatment effect over time	Small numbers, wide CIs. Most improvement during antibiotic treatment as in verified disc infection. Small changes in placebo group difficult to evaluate; might relate to un-blinding, see below
Scatterplot of change in RMDQ score	Change in RMDQ score at 1 year varied a lot within STIR3 amoxicillin group, indicating inconsistent subgroup effect
Bangs blinding index	In amoxicillin group − 0.01 (no un-blinding); in placebo group 0.61, i.e. possible un-blinding, which may have impaired improvement—or lack of improvement may have caused high blinding index

The 11 criteria were defined by Sun et al [34]

*STIR*, short tau inversion recovery; *RMDQ*, Rolland-Morris Disability Index; *CI*, confidence interval; *ODI*, Oswestry Disability Index; *LBP*, low back pain; *MCs*, Modic changes; *NNT*, number needed treat

Post hoc responder analyses supported an effect of amoxicillin in the STIR3 group, but the estimates showed wide CIs (Appendix, Tables A2–A3). Similar to patients with verified *Cutibacterium acnes* discitis [40], STIR3 patients improved most during antibiotic treatment (Fig. 4). The clinical course in the STIR3 placebo group is difficult to evaluate for credibility because the natural course in untreated groups with abundant MC-related oedema on STIR is unknown. The STIR3 placebo group reported almost no improvement during the 1-year follow-up (Fig. 4). Placebo groups and sick-listed patients with persistent LBP and type 1 MCs also reported little improvement over 1 year in some studies: 0.5–1.4 points for RMDQ [19, 41, 42], 1.9 points for ODI [43], and 0–2.2 points for LBP [19, 41–43].

Incomplete blinding may have contributed to lack of improvement in our placebo group and an overestimated effect of amoxicillin. All patients were blinded to treatment allocation, but placebo patients still tended to suspect they were not on active treatment (Table A4). This might be due to a lack of treatment effect or lack of side effects [44]. AIM patients with little improvement at 3 months and no side effects were less likely to report at 1 year that they had received antibiotics [21]. The precise impact of incomplete blinding on outcome is unclear [45].

As hypothesised, amoxicillin had the largest effect on RMDQ and ODI in the assumed ‘worst’ category of all STIR variables (Figs. 1 and 2). This was not the case in the type 1 major category on T1/T2, and the effect of placebo in the group ‘MC volume <10%’ is difficult to explain and may be spurious (Fig. 1). Thus, the results for STIR variables appear more credible than the explorative T1/T2 results. Below, we further discuss the STIR3 results that correspond to our predefined primary hypothesis.

### STIR3 results—interpretation and implications

The effect of amoxicillin was larger for STIR3 patients than for the original type 1 and type 2 only MC groups (− 2.3 and − 0.1 RMDQ points, respectively) in the primary analysis of AIM [19]. The disability at baseline was similar, not worse in the STIR3 group as expected, and cannot explain the difference. These findings make it relevant to examine patients with extensive MC-related oedema on STIR as a separate subgroup in future treatment studies.

It remains unclear why the treatment effect (RMDQ difference) at 1 year was smaller in the STIR3 group (5.1; 0–24 scale) than in the prior cohort with type 1 MCs (8.3; 0–23 scale) [19]. Baseline MC oedema cannot be compared because the prior trial applied 0.2-T MRI without STIR. Both cohorts had MCs at the level of a prior disc herniation. STIR3 patients had slightly lower baseline RMDQ scores than the previous cohort, less than 13 vs. 15 [19]; baseline LBP scores were similar, above 6.

The STIR3 results were consistent with the hypothesis that some MCs with abundant oedema on STIR might represent low-grade discitis. Importantly, spondylodiscitis was an exclusion criterion and was not suspected on MRI. The STIR3 findings were credible by most of the predefined criteria (Table 4) and the treatment course mirrored that of *Cutibacterium acnes* discitis.

However, replication of our findings is essential. The effect of amoxicillin in the STIR3 group varied greatly (Fig. 5) and was not evident for LBP intensity. The CI overlapped with the cut-off for clinical importance, bacterial infection was not verified, and un-blinding may have occurred. Additionally, adverse events and antibiotic resistance are potential harms [21].

The present findings support the use of STIR to evaluate MCs. They also motivate further studies of MC-related oedema on STIR in relation to possible biological markers of infection that are currently being investigated by our research group. To achieve an optimal classification of MCs for potential clinical use, MC characteristics not studied here, such as diffusion parameters [46], contrast enhancement, and bone turnover [6], should also be investigated.

Further work is needed to quantify STIR findings. The composite STIR variable was based on both visual assessments and several time-consuming manual measurements. The visually estimated STIR signal volume alone yielded similar results and might be more applicable in a clinical setting. However, precise measurements are preferable in research and may become more feasible with advanced automated techniques [47, 48]. To date, few studies have quantified spinal oedema on MRI [29, 49–51].

### Strengths and limitations

The strengths of this study include predefined hypotheses, standardised MRI techniques, and MRI ratings by three experienced radiologists [52]. Additionally, potential effect modifiers were defined and categorised before analysing their modifying impact.

Subgroup studies of clinical trials often have limited statistical power and generalisability [36, 53, 54]. This phenomenon also applies to our study. Our pre-decision in the statistical analysis plan to perform primary PP analyses is debatable, although we also present secondary ITT results. The decision implied that we focused primarily on the effect of amoxicillin and secondarily on the effect of allocating patients to receive amoxicillin [55, 56]. The impact of MRI findings on the treatment effect in patients who did not follow their assigned treatment seemed less relevant to study. PP analyses can create prognostic differences between the treatment groups [55]. However, ITT analyses supported the PP findings.

We evaluated the MRIs at inclusion before defining the subgroups, and the definitions of the MRI subgroups were partly dependent on the MRI data [57]. Furthermore, the composite MRI variables had not been validated. The ratings of STIR signal height and volume were less reliable at L5/S1 inferior to the disc [33]. However, the conclusive rating based on multiple observers’ evaluations was likely more reliable than each observer’s rating [52]. When an MC contained both type 1 and another type, we classified the type 1 part as primary or secondary, but we did not measure its exact size or intensity. Our results may not apply to low-field or 3-T MRI. However, they are likely valid with similar 1.5-T MRI protocols [3] and STIR works well with both low- and high-field scanners [58, 59].

## Conclusion

Predefined subgroups with chronic LBP, an index level with prior disc herniation, and abundant MC-related index-level oedema on STIR modified the treatment effect of amoxicillin. This finding shows moderate credibility based on published criteria and post hoc analyses and requires replication.

## Supplementary information

ESM 1 (DOCX 683 kb)

## Abbreviations

AIM: Antibiotics in Modic changes
CI: Confidence interval
ITT: Intention to treat
LBP: Low back pain
MCs: Modic changes
MRI: Magnetic resonance imaging
ODI: Oswestry Disability Index
PP: Per protocol
RMDQ: Roland-Morris Disability Questionnaire
STIR: Short tau inversion recovery
T1/T2: T1- and T2-weighted fast spin echo images
