# Supplementary material for: Oedema on STIR modified the effect of amoxicillin as treatment for chronic low back pain with Modic changes—subgroup analysis of a randomized trial
Source: Eur Radiol. 2020 Nov 27;31(6):4285–97. doi: 10.1007/s00330-020-07542-w (PMC8128743; doi:10.1007/s00330-020-07542-w)
Supplement: Supplementary file 1 — (DOCX 683 kb) [file 330_2020_7542_MOESM1_ESM.docx]

**Appendix**

**Oedema on STIR modified the effect of amoxicillin as treatment for chronic low back pain with Modic changes – subgroup analysis of a randomized trial**

**Table A1 Inclusion and exclusion criteria for the AIM- (Antibiotics In Modic changes) trial**

| **Inclusion criteria** | **Exclusion criteria** |
| --- | --- |
| - Age between 18 and 65 years - Low back pain (LBP) of more than 6 months duration in the area below the 12th rib and above the gluteal folds with a Numerical Rating Scale (NRS) pain intensity score of ≥5 (mean of three 0–10 NRSs: current LBP, the worst LBP within the last 2 weeks, and the usual/ mean LBP within the last 2 weeks) - MRI-confirmed lumbar disc herniation within the preceding 2 years - Type 1 and/or type 2 MC in the vertebral body marrow at the same level as the previously herniated disc. For patients with previous surgery for disc herniation, the MC has to be located at an operated level - Written informed consent | - Allergy to penicillin or cephalosporins - Allergy/hypersensitivity to any of the excipients of the study drug - Current pregnancy or lactation - Kidney (creatinine) or hepatic (ALAT/ASAT) laboratory values above the normal range - Phenylketonuria (Følling’s disease) - Mononucleosis or leukaemia - Any specific diagnosis that may explain the patient’s low back symptoms (e.g., tumour, fracture, spondyloarthritis, infection, spinal stenosis) - Previous low back surgery (L1–S1) for reasons other than disc herniation (e.g., fusion, decompression, disc prosthesis) - Surgery for disc herniation within the last 12 months - Previous surgery for disc herniation, but MC located at level(s) that has/have not been operated on only - Reservation about the intake of gelatine (the capsules used to encapsulate the study medicine contains gelatine, which, among other things, is produced using ingredients derived from pigs) - Regular use of glucocorticoids - Regular use of opioids with the exception of codeine and tramadol - Not understanding Norwegian language - Unlikely to adhere to treatment and/or complete follow-up (e.g., serious ongoing psychiatric disease, drug abuse, plans to move) - Antibiotic treatment within the preceding one month before treatment start - Contraindications to MRI (e.g., cardiac pacemaker electrodes, metal implant in the eye or brain, claustrophobia) - Unwilling to participate |

**Description of the per protocol population (155 of 180 randomized patients)**

The per protocol population consisted of all patients who completed the trial without major protocol deviations, defined as:

(a) intake of <80% of the pills (amoxicillin or placebo),

(b) pause of the study medication for ≥2 weeks (in the antibiotic group: without other ‘relevant’ antibiotic treatment in that period, i.e. treatment likely to affect *a Cutibacterium acnes* discitis),

(c) ‘relevant’ antibiotic treatment in the placebo group for ≥4 continuous weeks between baseline and one-year follow-up, and

(d) back surgery during the one-year follow-up.

Further events registered as major protocol deviations were incorrect enrolment (2 patients treated with antibiotics last month prior to inclusion), both amoxicillin and placebo given to patient by mistake (1 patient), and spondyloarthritis diagnosed during follow-up (1 patient).

See summary in the table below.

| **Amoxicillin group** | **Placebo group** |
| --- | --- |
| **89** Randomized (intention to treat population) | **91** Randomized (intention to treat population) |
| **85** Reported RMDQ score at 1 year | **84** Reported RMDQ score at 1 year |
| **77** Completed trial without major protocol deviations (**per protocol population**) | **78** Completed trial without major protocol deviations (**per protocol population**) |
|  |  |
| **3** End of study before 3 months | **2** End of study before 3 months |
| **7** Treatment non-completion | **8** Treatment non-completion |
| **1** Operation for disc herniation | **1** Diagnosed as having spondyloarthritis |
| **1** Incorrect enrolment | **1** Given both amoxicillin and placebo |
|  | **1** Incorrect enrolment |
| RMDQ= Rolland-Morris Disability Questionnaire | |

**Figure A1 Rolland-Morris Disability Questionnaire (RMDQ) for all effect modifiers (intention to treat)**


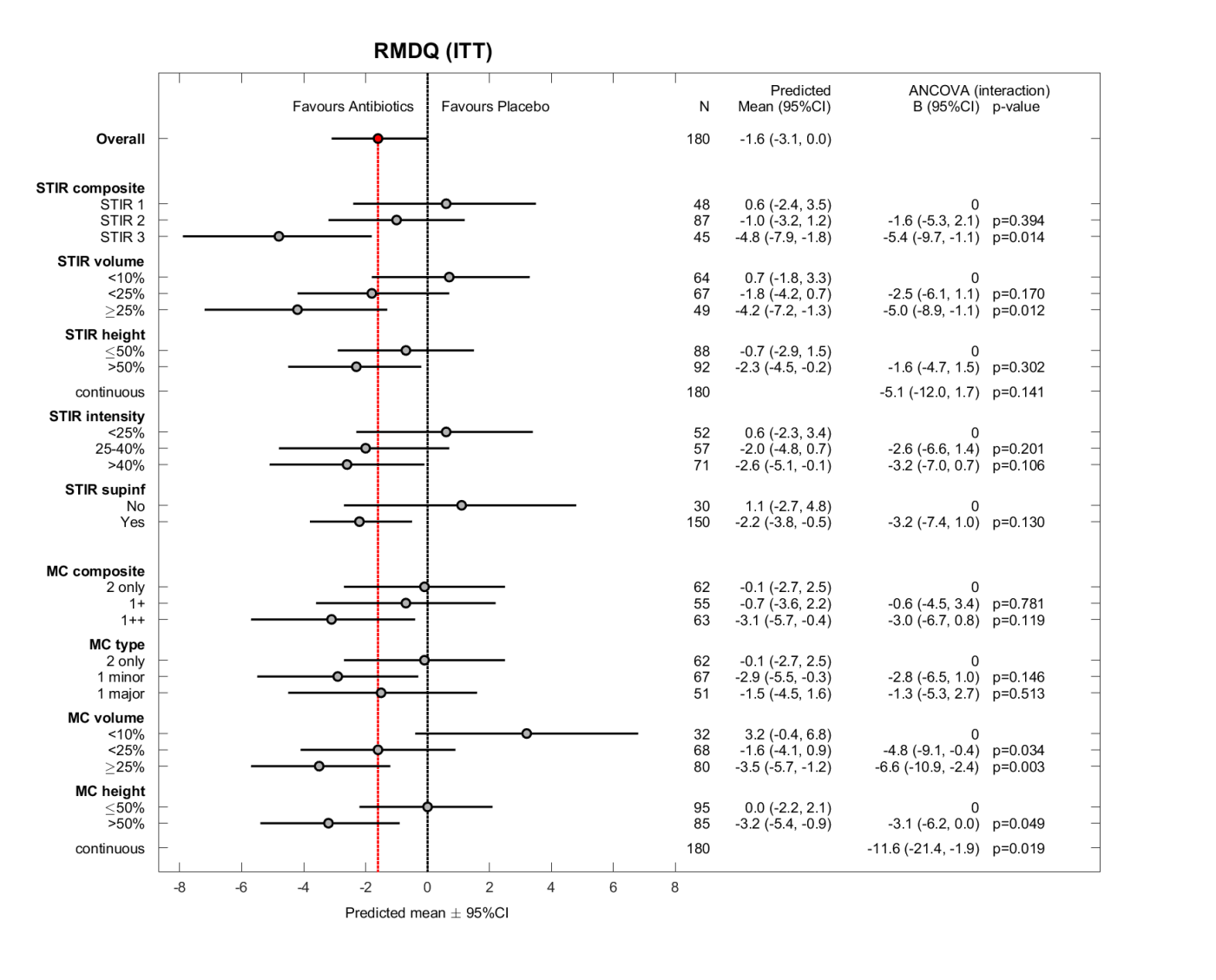


RMDQ scores range from 0 (no disability) to 24 (maximum disability). Observed difference between treatment groups (Mean ± 95%CI) and estimated coefficients (with 95%CI) for interaction from the ANCOVA (intention to treat) with p-values.

ITT=intention to treat. CI=confidence interval. STIR=short tau inversion recovery. MC=Modic change. MC variables are based on T1- and T2-weighted fast spin echo images, not STIR.

**Figure A2 Oswestry Disability Index (ODI) for all effect modifiers (intention to treat)**

**
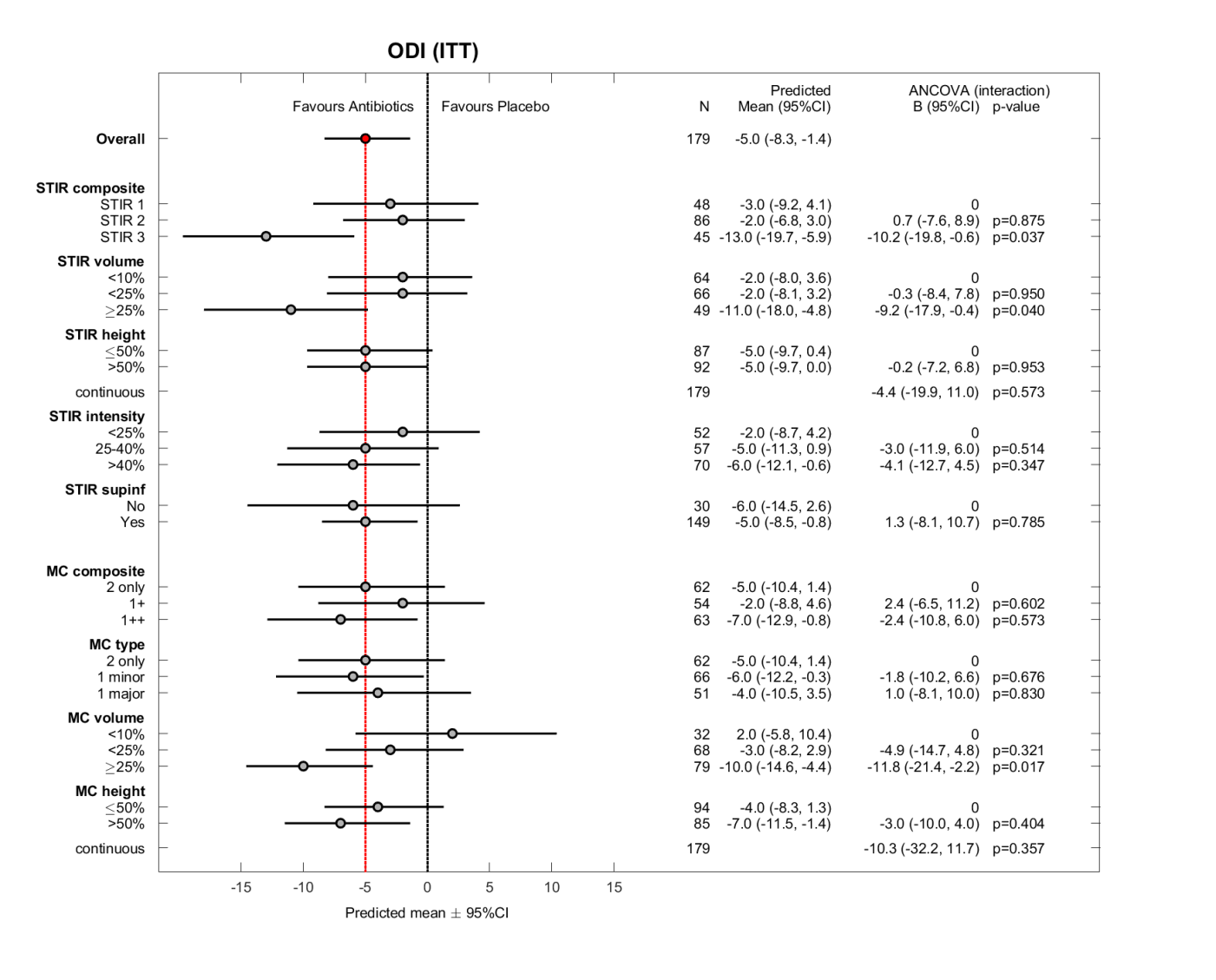
**

ODI scores range from 0 (no disability) to 100 (maximum disability). Observed difference between treatment groups (Mean ± 95%CI) and estimated coefficients (with 95%CI) for interaction from the ANCOVA (intention to treat) with p-values. Missing value not imputed in one patient (excluded).

ITT=intention to treat. CI=confidence interval. STIR=short tau inversion recovery. MC=Modic change. MC variables are based on T1- and T2-weighted fast spin echo images, not STIR.

**Figure A3 Low back pain intensity for all effect modifiers (intention to treat)**

**
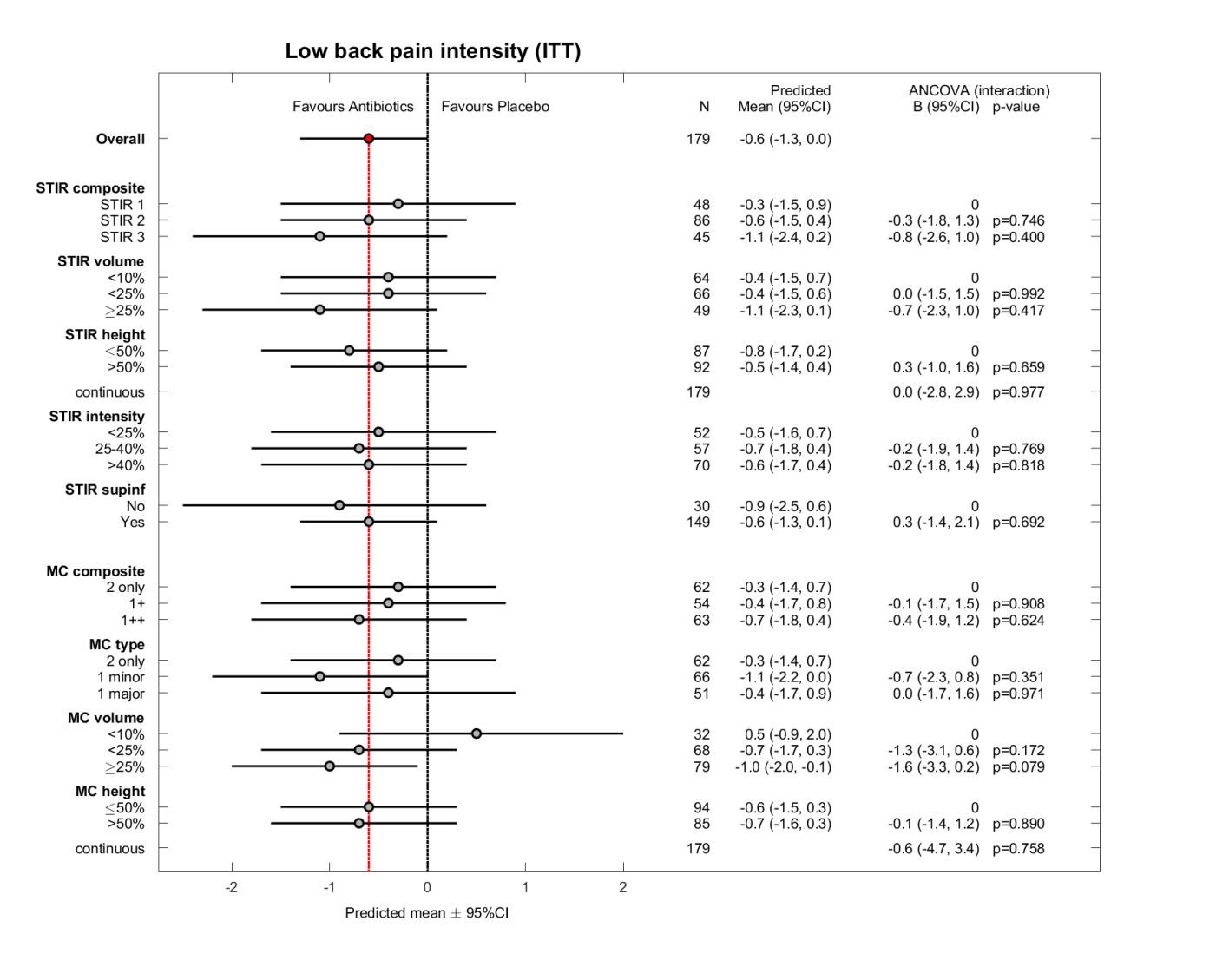
**

Pain intensity scores range from 0 (no pain) to 10 (worst possible pain). Observed difference between treatment groups (Mean ± 95%CI) and estimated coefficients (with 95%CI) for interaction from the ANCOVA (intention to treat) with p-values. Missing value not imputed in one patient (excluded).

ITT=intention to treat. CI=confidence interval. STIR=short tau inversion recovery. MC=Modic change. MC variables are based on T1- and T2-weighted fast spin echo images, not STIR.

| **Table A2 Responders among STIR3 patients randomized to amoxicillin or placebo** | | | | | |
| --- | --- | --- | --- | --- | --- |
| Improvement in RMDQ score from baseline to one year | | Amoxicillin^a^ n/total n (%) | Placebo n/total n (%) | NNT (95% CI) | P-value^b^ |
| Per protocol population | |  |  |  |  |
|  | Improved >30% | 12/21 (59) | 5/20 (25) | 3.1 (1.7 to 27) | 0.04 |
|  | Improved >50% | 10/21 (48) | 4/20 (20) | 3.6 (1.8 to -7044) | 0.06 |
|  | Improved >75% | 6/21 (29) | 1/20 (5) | 4.2 (2.2 to 50) | 0.045 |
| Intention to treat population | |  |  |  |  |
|  | Improved >30% | 12/22 (55) | 5/21 (24) | 3.3 (1.7 to 32) | 0.04 |
|  | Improved >50% | 10/22 (45) | 4/21 (19) | 3.8 (1.9 to -301) | 0.06 |
|  | Improved >75% | 6/22 (27) | 1/21 (5) | 4.4 (2.3 to 56) | 0.046 |
| Table shows results from post-hoc analyses. STIR=short tau inversion recovery. RMDQ=Rolland-Morris Disability Questionnaire. NNT= number needed to treat. CI=confidence interval.  ^a^Two patients with missing RMDQ scores not included. ^b^Chi-square test. | | | | | |

| **Table A3 Responders by STIR group for all patients with complete RMDQ data** | | | | |
| --- | --- | --- | --- | --- |
| Improvement in RMDQ score at one year | STIR1  n/total n (%) | STIR2  n/total n (%) | STIR3  n/total n (%) | Total  n/total n (%) |
| Placebo, >30% | 5/22 (23) | 14/40 (35) | 5/21 (24) | 24/83 (29) |
| Placebo, >50% | 5/22 (23) | 9/40 (23) | 4/21 (19) | 18/83 (22) |
| Placebo, >75% | 2/22 (9) | 4/40 (10) | 1/21 (5) | 7/83 (8) |
|  |  |  |  |  |
| Amoxicillin, >30% | 9/21 (43) | 19/41 (46) | 12/22 (55) | 40/84 (48) |
| Amoxicillin, >50% | 1/21 (5) | 12/41 (29) | 10/22 (45) | 23/84 (27) |
| Amoxicillin, >75% | 0/21 (0) | 9/41 (22) | 6/22 (27) | 15/84 (18) |
|  |  |  |  |  |
| Placebo, RMDQ missing | 3/25 (12) | 5/45 (11) | 0/21 (0) | 8/91 (9) |
| Amoxicillin, RMDQ missing | 2/23 (9) | 1/42 (2) | 2/24 (8) | 5/89 (6) |
| Table shows results from post-hoc analyses. STIR=short tau inversion recovery.  RMDQ=Rolland-Morris Disability Questionnaire. | | | | |

| **Table A4 Response to blinding question^a^ at one year from STIR3 patients in the PP population** | | |
| --- | --- | --- |
|  | Amoxicillin group (n=21) | Placebo group (n=20) |
| Antibiotics | 7 | 1 |
| Placebo | 7 | 12 |
| Unsure | 7 | 7 |
| Unsure - antibiotics | 3 | 1 |
| Unsure - placebo | 4 | 6 |
| Bangs blinding index^b^ (95% CI) | -0.01 (-0.31 to 0.29) | 0.61 (0.41 to 0.81) |
| Table shows results from post-hoc analyses. PP=per protocol. CI=confidence interval.  ^a^Which study medicine do you think you received? (antibiotics / placebo / unsure; if unsure, which one is most likely). ^b^Bangs blinding index [1] ranges from −1 (all report incorrect treatment) to 1 (all report correct treatment); 0=random reporting of treatment group. | | |

| **Table A5 Characteristics of STIR3 patients. Values are n/total n (%) unless stated otherwise** | | |
| --- | --- | --- |
|  | Amoxicillin group (n=24) | Placebo group (n=21) |
| **Per protocol population** | 21/24 (88) | 20/21 (95) |
| **Baseline characteristics** |  |  |
| Age, mean (SD) | 43 (10) | 46 (9) |
| Women | 13/24 (54) | 15/21 (71) |
| Previous disc herniation surgery | 2/24 (8) | 5/21 (24) |
| Body mass index, mean (SD) | 26 (4.2) | 25 (3.8) |
| Smoking, yes | 10/24 (42) | 8/21 (38) |
| Educational level |  |  |
| -Primary school (9 years) | 6/24 (25) | 2/21 (10) |
| -High school (12 years) | 6/24 (25) | 7/21 (33) |
| -College or university (<4 years) | 11/24 (46) | 7/21 (33) |
| -University (≥4 years) | 1/24 (4) | 5/21 (24) |
| Comorbidity^a^ |  |  |
| -Score 1 (back pain only) | 18/24 (75) | 16/21 (76) |
| -Score 2 | 2/24 (8) | 4/21 (19) |
| -Score >2 | 4/24 (17) | 1/21 (5) |
| Emotional distress^b^ (HSCL-25 ≥1.75) | 3/24 (13) | 4/21 (19) |
| FABQ physical activity^c^ (0-24), mean (SD) | 11 (6) | 14 (5) |
| FABQ work^c^ (0-24), mean (SD) | 17 (13) | 19 (12) |
| Duration of back pain (years), median (IQR) | 3 (1.5-5.4) | 2.8 (2-4.5) |
| Physical workload (data missing in 4 patients) |  |  |
| -Mostly sitting | 9/21 (43) | 7/20 (35) |
| -Job requires a lot of walking | 5/21 (24) | 4/20 (20) |
| -Job requires a lot of walking and lifting | 7/21 (33) | 7/20 (35) |
| -Job requires physically heavy work | 0/21 (0) | 2/20 (10) |
| Employment status |  |  |
| -Working full time | 14/24 (58) | 13/21 (62) |
| -Partial sick leave | 1/24 (4) | 3/21 (14) |
| -Complete sick leave | 7/24 (29) | 5/21 (24) |
| -Disability pension | 1/24 (4) | 0/21 (0) |
| -Unemployed | 1/24 (4) | 0/21 (0) |
| RMDQ score (0-24), mean (SD) | 12.8 (4.4) | 12.6 (4.0) |
| ODI score (0-100), mean (SD) | 31.1 (9.5) | 28.3 (9.3) |
| Low back pain intensity (0-10), mean (SD) | 6.3 (1.1) | 6.4 (1.4) |
| Presence of leg pain | 18/24 (75) | 16/21 (76) |
| Leg pain intensity (0-10), median (IQR) | 2 (0.5-5) | 2 (1-3) |
| Pre-study expectations of antibiotic treatment |  |  |
| -fully cured back pain | 5/24 (21) | 5/21 (24) |
| -large improvement | 11/24 (46) | 11/21 (52) |
| -slight improvement | 2/24 (8) | 0/21 (0) |
| -no improvement | 0/24 (0) | 0/21 (0) |
| -don’t know | 6/24 (25) | 5/21 (24) |
| Index level(s) with MC and prior disc herniation |  |  |
| -L3/L4 | 3/24 (13) | 1/21 (5) |
| -L4/L5 | 15/24 (63) | 10/21 (48) |
| -L5/S1 | 11/24 (46) | 16/21 (76) |
| MC type 1 group | 20/24 (83) | 18/21 (86) |
| **Concomitant therapy,** physical / manual |  |  |
| -at baseline, yes | 5/24 (21) | 2/21 (10) |
| -month 1-3 of study, yes | 4/24 (17) | 2/21 (10) |
| -month 1-3, months of therapy, mean | 6/24 = 0.25 | 6/21 = 0.29 |
| -month 4-12 of study, yes | 3/24 (13) | 7/21 (33) |
| -month 4-12, months of therapy, mean | 15/24 = 0.63 | 15/21 = 0.71 |
| -at baseline or during month 1-12, yes | 8/24 (33) | 8/21 (38) |
| Table shows results from post-hoc analyses. MC=Modic change. SD=standard deviation. IQR=interquartile range. RMDQ=Rolland-Morris Disability Questionnaire. ODI=Oswestry Disability Index.  ^a^Functional Comorbidity Index [2]; score increased by 1 for each of 18 diagnoses associated with decreased physical function. ^b^Hopkins Symptom Checklist-25 [3]; values ≥1.75 related to psychiatric diagnosis. ^c^Fear Avoidance Beliefs Questionnaire [4]; higher values indicate more fear avoidance beliefs. | | |

**Comment to Table A5**

In ANCOVA, we adjusted for age, prior disc herniation surgery, and baseline values of the outcome. Post-hoc, we compared known predictors for low back pain [5, 6] between STIR3 treatment groups (without statistical testing) to assess if any of these variables should also be adjusted for when performing ANCOVA to assess the subgroup effect of STIR3. These additional variables (all predefined in the Statistical analysis plan for AIM) were comorbidity, emotional distress, fear-avoidance beliefs, physical workload, employment status, low back pain intensity, and leg pain intensity (**Table A5**).

We also compared all other baseline factors in **Table A5**, including treatment expectations [7], between STIR3 treatment groups. **Table A5** shows characteristics of all STIR3 patients. STIR3 patients in the per-protocol population had similar distribution of characteristics between treatment groups.

The Amoxicillin group tended to have less education and more comorbidity (can worsen prognosis), but less physical workload (can improve prognosis). The placebo group tended to report greater pre-study expectations for effect of amoxicillin, but the difference was minimal.

We decided not to adjust for any of these minor imbalances, which partly outweighed each other.

The amoxicillin group tended to receive more physical / manual therapy at baseline and during the treatment period (month 1-3) and less thereafter (month 4-12). Numbers were small and concomitant therapy was similar between the treatment groups during the total study period including baseline.

**References**

1 Bang H, Ni L, Davis CE (2004) Assessment of blinding in clinical trials. Control Clin Trials 25:143-156

2 Groll DL, To T, Bombardier C, Wright JG (2005) The development of a comorbidity index with physical function as the outcome. J Clin Epidemiol 58:595-602

3 Sandanger I, Moum T, Ingebrigtsen G, Dalgard OS, Sorensen T, Bruusgaard D (1998) Concordance between symptom screening and diagnostic procedure: the Hopkins Symptom Checklist-25 and the Composite International Diagnostic Interview I. Soc Psychiatry Psychiatr Epidemiol 33:345-354

4 Waddell G, Newton M, Henderson I, Somerville D, Main CJ (1993) A Fear-Avoidance Beliefs Questionnaire (FABQ) and the role of fear-avoidance beliefs in chronic low back pain and disability. Pain 52:157-168

5 Hartvigsen J, Hancock MJ, Kongsted A et al (2018) What low back pain is and why we need to pay attention. Lancet 391:2356-2367

6 Hayden JA, Chou R, Hogg-Johnson S, Bombardier C (2009) Systematic reviews of low back pain prognosis had variable methods and results: guidance for future prognosis reviews. J Clin Epidemiol 62:781-796 e781

7 Hayden JA, Wilson MN, Riley RD, Iles R, Pincus T, Ogilvie R (2019) Individual recovery expectations and prognosis of outcomes in non-specific low back pain: prognostic factor review. Cochrane Database Syst Rev 2019
